# Supplementary material for: COZOID: contact zone identifier for visual analysis of protein-protein interactions
Source: BMC Bioinformatics. 2018 Apr 6;19:125. doi: 10.1186/s12859-018-2113-6 (PMC5889581; doi:10.1186/s12859-018-2113-6)
Supplement: Supplementary file 2 — Software build. Executable binary file of the software tool. (ZIP 118,784 kb) [file 12859_2018_2113_MOESM2_ESM.zip › cozoid/java/docs/beansbinding-1.2.1-doc.zip!/org/jdesktop/swingbinding/JTableBinding.ColumnBinding.html]

JTableBinding.ColumnBinding (Beans Binding)


---


|  |  |  |
| --- | --- | --- |
| **Overview** \| **Package** \| **Class** \| **Use** \| **Tree** \| **Deprecated** \| **Index** \| **Help** | |  |
| **PREV CLASS**   **NEXT CLASS** | **FRAMES**    **NO FRAMES**     **All Classes** |  |
| SUMMARY: NESTED \| FIELD \| CONSTR \| METHOD | DETAIL: FIELD \| CONSTR \| METHOD |  |


---


## org.jdesktop.swingbinding Class JTableBinding.ColumnBinding

```
java.lang.Object
  org.jdesktop.beansbinding.Binding
      org.jdesktop.swingbinding.impl.AbstractColumnBinding
          org.jdesktop.swingbinding.JTableBinding.ColumnBinding
```

**Enclosing class:**: JTableBinding<E,SS,TS>

---

``` public final class JTableBinding.ColumnBinding extends AbstractColumnBinding ```

`ColumnBinding` represents a binding between a property of the elements
in the `JTableBinding's` source `List`, and a column in the table. Each
`ColumnBinding` added to a `JTableBinding` represents a column
to be displayed by the `JTable`. A value for any given row in a column
is aquired by fetching the value of the associated `ColumnBinding's`
source property for the element in the source `List` representing that row.

A `Converter` may be specified on a `ColumnBinding`, as may be
a `Validator`. Validation occurs at the time a cell value is to be
committed back to the source `List`.

`BindingListeners` registered on
a `ColumnBinding` are notified of successful `sync` or
`syncFailure`. These notifications are also sent to the
`JTableBinding's` `BindingListeners`.

`ColumnBindings` are managed by their `JTableBinding`. They are not
to be explicitly bound, unbound, added to a `BindingGroup`, or accessed
in a way that is not allowed for a managed binding.

**See Also:**: `JTableBinding.addColumnBinding(Property, String)`

---

**Nested Class Summary**

**Nested classes/interfaces inherited from class org.jdesktop.beansbinding.Binding**

`Binding.SyncFailure, Binding.SyncFailureType, Binding.ValueResult<V>`


| **Method Summary** | |
| --- | --- |
| `java.lang.Class<?>` | `getColumnClass()`             Returns the column class to be used by `JTable` to determine the renderer and editor for the column represented by this `ColumnBinding`. |
| `java.lang.String` | `getColumnName()`             Returns the name for the column represented by this `ColumnBinding`. |
| `boolean` | `isEditable()`             Returns whether or not the cells of the column should be editable. |
| `JTableBinding.ColumnBinding` | `setColumnClass(java.lang.Class<?> columnClass)`             Sets the column class to be used by `JTable` to determine the renderer and editor for the column represented by this `ColumnBinding`. |
| `JTableBinding.ColumnBinding` | `setColumnName(java.lang.String name)`             Sets a name for the column represented by this `ColumnBinding`. |
| `JTableBinding.ColumnBinding` | `setEditable(boolean editable)`             Sets whether or not the cells of the column should be editable. |

**Methods inherited from class org.jdesktop.swingbinding.impl.AbstractColumnBinding**

`bindImpl, getColumn, setColumn, unbindImpl`

**Methods inherited from class org.jdesktop.beansbinding.Binding**

`addBindingListener, addPropertyChangeListener, addPropertyChangeListener, bind, bindUnmanaged, firePropertyChange, getBindingListeners, getConverter, getName, getPropertyChangeListeners, getPropertyChangeListeners, getSourceNullValue, getSourceObject, getSourceProperty, getSourceUnreadableValue, getSourceValueForTarget, getTargetNullValue, getTargetObject, getTargetProperty, getTargetValueForSource, getValidator, isBound, isManaged, isSourceUnreadableValueSet, notifySynced, notifySyncFailed, paramString, refresh, refreshAndNotify, refreshAndNotifyUnmanaged, refreshUnmanaged, removeBindingListener, removePropertyChangeListener, removePropertyChangeListener, save, saveAndNotify, saveAndNotifyUnmanaged, saveUnmanaged, setConverter, setManaged, setSourceNullValue, setSourceObject, setSourceObjectUnmanaged, setSourceProperty, setSourceUnreadableValue, setTargetNullValue, setTargetObject, setTargetObjectUnmanaged, setTargetProperty, setValidator, sourceChangedImpl, targetChangedImpl, throwIfBound, throwIfManaged, throwIfUnbound, toString, unbind, unbindUnmanaged, unsetSourceUnreadableValue`

**Methods inherited from class java.lang.Object**

`clone, equals, finalize, getClass, hashCode, notify, notifyAll, wait, wait, wait`

**Method Detail**

### setColumnName

```
public JTableBinding.ColumnBinding setColumnName(java.lang.String name)
```

:   Sets a name for the column represented by this `ColumnBinding`.
    This is used to initialize the table's column header name. If
    `null` is specified, the `toString()` value of the
    `ColumnBinding's` source property is used.

    :   **Parameters:**: `name` - the name **Returns:**: the `ColumnBinding` itself, to allow for method chaining **See Also:**: `TableModel.getColumnName(int)`

---


### setColumnClass

```
public JTableBinding.ColumnBinding setColumnClass(java.lang.Class<?> columnClass)
```

:   Sets the column class to be used by `JTable` to determine
    the renderer and editor for the column represented by this
    `ColumnBinding`.

    :   **Parameters:**: `columnClass` - the column class **Returns:**: the `ColumnBinding` itself, to allow for method chaining **See Also:**: `TableModel.getColumnClass(int)`

---


### getColumnClass

```
public java.lang.Class<?> getColumnClass()
```

:   Returns the column class to be used by `JTable` to determine
    the renderer and editor for the column represented by this
    `ColumnBinding`.

    :   **See Also:**: `setColumnClass(java.lang.Class )`, `TableModel.getColumnClass(int)`

---


### getColumnName

```
public java.lang.String getColumnName()
```

:   Returns the name for the column represented by this `ColumnBinding`.
    This is used to initialize the table's column header name. If no name
    has been specified, or if it has been set to `null`, the
    `toString()` value of the `ColumnBinding's` source property is returned.

    :   **Returns:**: the name for the column **See Also:**: `setColumnName(java.lang.String)`, `TableModel.getColumnName(int)`

---


### setEditable

```
public JTableBinding.ColumnBinding setEditable(boolean editable)
```

:   Sets whether or not the cells of the column should be editable.
    The default for this property is `true`.
    See this paragraph in the class level
    documentation on editability.

    :   **Parameters:**: `editable` - whether or not the cells of the column should be editable **Returns:**: the `ColumnBinding` itself, to allow for method chaining

---


### isEditable

```
public boolean isEditable()
```

:   Returns whether or not the cells of the column should be editable.
    The default for this property is `true`.
    See this paragraph in the class level
    documentation on editability.

    :   **Returns:**: whether or not the cells of the column should be editable


---


|  |  |  |
| --- | --- | --- |
| **Overview** \| **Package** \| **Class** \| **Use** \| **Tree** \| **Deprecated** \| **Index** \| **Help** | |  |
| **PREV CLASS**   **NEXT CLASS** | **FRAMES**    **NO FRAMES**     **All Classes** |  |
| SUMMARY: NESTED \| FIELD \| CONSTR \| METHOD | DETAIL: FIELD \| CONSTR \| METHOD |  |


---
